# Deception as a Derived Function of Language

**DOI:** 10.3389/fpsyg.2016.01485

**Published:** 2016-09-27

**Authors:** Nathan Oesch

**Affiliations:** Department of Experimental Psychology, University of OxfordOxford, UK

**Keywords:** spoken language, language evolution, language origins, direct and derived functions, vocal behavior, deception, manipulation

## Abstract

Language may be one of most important attributes which separates humans from other animal species. It has been suggested by some commentators that the primary biological function of human language is to deceive and selfishly manipulate social competitors. However, despite the existence of a large body of relevant theoretical and empirical literature in favor of the social bonding hypothesis for language function, the ostensible evidence and arguments for the deception hypothesis have not been fully discussed. The following review analyses the evidence and theoretical arguments from human social behavior, comparative animal behavior, and developmental psychology and suggests that deception shows clear signs of a derived function for language. Furthermore, in addition to being used relatively infrequently across most human and non-human animal contexts, deception appears to be utilized just as often for prosocial and social bonding functions, as it is for antisocial purposes. Future studies should focus on theoretical and experimental investigations which explore interactions between deceptive and honest language use in the context of social bonding.

## Introduction

Language may be the single most important feature which differentiates humans from other animal species (Christiansen and Kirby, 2003; Fitch, 2010). Although, many non-human animals have evolved the ability to communicate (Searcy and Nowicki, 2005; Bradbury and Vehrencamp, 2011), no other organisms aside from humans can take a finite number of components – more than 10,000 words for the typical adult language user (Goulden et al., 1990; D’Anna et al., 1991; Zechmeister et al., 1995; Treffers-Daller and Milton, 2013) – and link them together into a vast array of potential combinations, all of which hold distinct meanings (Pinker, 1997). Moreover, language may be integral in supplementing humans’ ability to engage in advanced forms of social cognition and understanding others’ intentions, including capacities such as Theory of Mind and higher-order intentionality (Dunbar, 1998, 2009; Cheney and Seyfarth, 2007).

Many biological functions for language have been postulated, including cultural learning (Pinker and Bloom, 1990), social bonding (Dunbar, 2004), and deception (Scott-Phillips, 2006). In most cases, such proposals often stress the importance of one primary functional candidate, and occasionally posit other purposes as additional secondary uses for language (De Backer and Gurven, 2006; Galantucci and Roberts, 2014). In the context of language evolution, this distinction is often referred to as the difference between *direct* and *derived* functions (Millikan, 1984; Origgi and Sperber, 2000). Further, despite the existence of a large body of relevant theoretical and empirical research in favor of the social bonding hypothesis as a direct function, (Dunbar, 1993, 2003, 2004; Nettle and Dunbar, 1997; McComb and Semple, 2005; Mesoudi et al., 2006; Roberts, 2010, 2013; Ireland et al., 2011; Weaver and Bosson, 2011; Cohen, 2012; Freeberg et al., 2012; Laidre et al., 2012; Dávid-Barrett and Dunbar, 2013; Redhead and Dunbar, 2013; Galantucci and Roberts, 2014; Pietraszewski and Schwartz, 2014), there is nonetheless still considerable debate over the issue as to how and why language originated exclusively in the human species (Christiansen and Kirby, 2003; Számadó and Szathmáry, 2006; Larson et al., 2010).

Impressed with the apparent ubiquity of deception found throughout the animal kingdom, some have argued that most animal communication systems have evolved primarily (as opposed to secondarily as a derived function) for the function of deception (Dawkins and Krebs, 1978; Krebs and Dawkins, 1984; Scott-Phillips, 2006). According to this hypothesis, the speaker (or signaller) may benefit from the breakdown of the normative relationship between speaker attribute or referent and the actual nature of the external world, while the listener (or receiver) may incur a significant cost from this misrepresentation (Searcy and Nowicki, 2005). For instance, in a classic series of articles by Dawkins and Krebs (1978) and Krebs and Dawkins (1984), it was argued that animal signaling and by extension human language, ought to be viewed as an evolutionary arms race in which signallers evolve to become better at manipulating receivers, while receivers evolve to become more resistant to manipulation. Granted, although manipulation can in principle be thought of in cooperative terms, and need not always imply competition, this particular conceptualization is apparently intended to refer fundamentally to adversarial relationships (Dawkins and Krebs, 1978; Krebs and Dawkins, 1984). According to this perspective, animal communication is defined solely in terms of the way in which signaling generates functional consequences primarily for the signaller: “communication is said to occur when an animal, the actor, does something to influence the sense organs of another animal, the reactor, so that the reactor’s behavior changes to the advantage of the actor” (Dawkins and Krebs, 1978, p. 283). Additionally, at least one evolutionary linguist has voiced a very similar assertion, in that human language evolved primarily for the function of selfish signaller mind-reading and manipulation (Scott-Phillips, 2006). According to this hypothesis, human language is viewed as a fundamentally deceptive method of communication.

However, currently available evidence suggests language more likely directly evolved for social bonding functions, while secondarily derived for other purposes (De Backer and Gurven, 2006; Galantucci and Roberts, 2014), including deception. To this end, the following review analyses available evidence from human social behavior, comparative animal behavior, and developmental psychology which point to the relative paucity and rareness of deception found in these relevant contexts. Further, as language is a behavior and does not fossilize, speculative accounts that invoke archeological claims as evidence, as is often done by many theorists (Számadó, 2010; Bickerton and Szathmáry, 2011), will be avoided. Ultimately, a careful and thorough analysis of language and animal communication, when used for deceptive purposes, in fact reveals substantially more evidence in favor of social bonding as the primary function of language, while suggesting a derived function for deception.

For present purposes, deception is defined here as the phenomenon where a signaller benefits from the breakdown of the normative correlation between signaller characteristic and external attribute (Searcy and Nowicki, 2005). In more colloquial terms, deception has been defined elsewhere as “the projection, to one’s own advantage, of an inaccurate or false image of knowledge, intentions, or motivations” (deWaal, 2005, p. 86). For purposes of clarification and consistency, language is further defined here as the uniquely human biological capacity for complex vocal imitation – the ability to imitate complex multisyllabic vocalizations – including the cognitive, anatomical, physiological, and genetic systems that underpin human speech (Fitch, 2010).

## Evidence for Deception as a Derived Function of Language

### Comparative Animal Behavior

The evolutionary strategy of deception, once thought unique to humans, has now been documented in a relatively small number of non-human primate species (Whiten and Byrne, 1988a; Byrne and Whiten, 1990; deWaal, 2005), including chimpanzees (Hare et al., 2006; Slocombe and Zuberbühler, 2007), baboons (Byrne and Whiten, 1985), rhesus (Gouzoules et al., 1996), macaque (Overduin-de Vries et al., 2015) and tufted capuchin monkeys (Wheeler, 2009), as well as birds (Munn, 1986; Møller, 1988, 1990; Tramer, 1994; Bugnyarf and Kotrschal, 2002; Igic et al., 2015), ungulates (Bro-Jørgensen and Pangle, 2010), foxes (Rüppell, 1986) and squirrels (Tamura, 1995; but see Cheney and Seyfarth, 1990 for an alternative account of deception in a greater variety of non-human primates). Chimpanzees, for instance, have been observed using deceptive non-verbal signals in the wild, as well as deceitful sign-language mimicry in trained conditions (Byrne and Whiten, 1990; deWaal, 2005; Kirkpatrick, 2007). This particular form of deceit, or *tactical deception*, is typically observed as the concealment of a thing, behavior or an emotion (Whiten and Byrne, 1988b; Byrne and Whiten, 1990; Hauser, 1997; Whiten, 1997). For example, in one well-documented case, a chimpanzee was approached from the rear by a noisily heard aggressive challenger: to conceal his fearful expression, the chimpanzee affected his lips and face in such a way as to rid himself of the fear grin, before turning to face his competitor (deWaal, 1986, p. 233; Byrne and Whiten, 1991, p. 134).

Further, there are even fewer cases of animal communication which might be aptly characterized as this sort of manipulative tactical deception, often referred to as *functional deception*, which more commonly occurs in humans (Byrne and Whiten, 1990; Talwar and Lee, 2008). Indeed, in highlighting the relatively paucity found throughout the animal kingdom, Byrne and Whiten (1990, p. 3) define tactical deception accordingly as “acts *from the normal* repertoire of [an] agent, deployed such that another individual is likely to misinterpret what the acts signify, to the advantage of the agent [italics added].” As such, this sort of deception can prove potentially costly to the user, as tactical deception mostly occurs in social animals, which may lose trust of fellow group-members when their deceit is discovered (Smith, 1986; Cheney and Seyfarth, 1990; deWaal, 2005).

In the vast majority of documented cases, tactical deception most frequently occurs in cases where alarm calls or warning cries are used deceptively to obtain access to increased food supplies or reproductive opportunities (Byrne and Whiten, 1985; Munn, 1986; Rüppell, 1986; Møller, 1988, 1990; Tramer, 1994; Tamura, 1995; Bugnyarf and Kotrschal, 2002; Bro-Jørgensen and Pangle, 2010). For instance, male topi antelopes have been found to alarm snort deceptively in order to retain receptive females within the dominant males’ territory, precluding them from straying too far from the group (Bro-Jørgensen and Pangle, 2010). Among primates, subordinate tufted capuchin monkeys have been found to deceptively use alarm calls (normally reserved for predator sightings) to evoke a response from other members of the group when competing with dominant monkeys over valuable food resources (in effect, taking advantage of the distraction and fleeing the scene in order to steal food; Wheeler, 2009). Similar findings have been reported in Amazonian birds, including the white-winged shrike tanager and the bluish-slate antshrike (Munn, 1986). In addition, observations of wild geladas have found that monkeys involved in surreptitious extra-pair copulations were actually *less* inclined to vocalize and more inclined to copulate when the cuckolded male was in distant proximity, as vocal deceivers typically receive significantly more physical aggression than non-vocal deceivers (le Roux et al., 2013). Similar acts of apparent punishment and aggression have been observed in rhesus monkeys who fail to vocalize their discovery of food to the rest of the group (Hauser, 1992; although see Jensen, 2010 for an alternative perspective).

Nonetheless, considerable evidence shows deception, at least in chimpanzees, more often occurs through non-vocal expression, gesture, and various body postures, indicating vocal communication is not always a necessary or even favored requirement (deWaal, 2005). Moreover, because the costs of deception are believed to be relatively high for the deceiver if discovered, tactical deception has thus far been documented in very few cases; as such, it is believed to be more common in cases where the costs of neglecting the possibly deceptive act are even higher than the costs of believing (e.g., ignoring the call which could potentially result in death by predation; Hauser, 1992; Wheeler, 2009). In summary, currently available evidence indicates, particularly in social group-living animals where trust is often important, the scarcity, potential risk, and highly context-sensitive nature of most acts of deception within animal communication (Smith, 1986; Cheney and Seyfarth, 1990; deWaal, 2005).

### Developmental Psychology

Further suggestions for a derived function for human deception have come from child development studies. For example, experimental studies examining children’s antisocial deception (i.e., lying to hurt or disbenefit someone) have found that self-serving lies (e.g., to conceal a transgression), typically occur between 4 and 7 years of age, with lies below this age range occurring much more infrequently, where the default seems to be honesty (Talwar and Lee, 2008; Lee, 2013). Nonetheless, while children seem, in most cases, more willing to lie for familiar others than strangers, self-interest seems to be a generally stronger motivator for deception (Talwar and Lee, 2008).

On the other hand, while non-human primates have been observed using tactical deception for self-interest, prosocial deception (i.e., lying to protect someone, or to benefit or help others), appears to be unique to humans (Talwar and Lee, 2008). In fact, studies have shown that older children beyond age 7 are generally more inclined to tell prosocial ‘white lies’ (i.e., lies intended to spare the feelings of the person being lied to), despite potential costs to themselves (Talwar and Lee, 2008; Lee, 2013). For example, in one noteworthy study, children were instructed to take the photo of a confederate experimenter with an unattractive mark on their face. In most cases, when the experimenter queried: “Do I look okay for the photo?,” the children lied, telling the experimenter that they looked fine, while later confiding to someone else that they actually didn’t look okay (Talwar and Lee, 2002). Moreover, as children grow older, they become more likely to be concerned with the feelings and needs of others, and therefore more likely to tell prosocial white lies, as well as prosocial ‘blue lies’ (i.e., lies intended to benefit an in-group collective; Talwar and Lee, 2008; Lee, 2013). For instance, in one study of blue lies, children were embedded in a context in which their class brazenly opted to break the rules when choosing team members to represent their class in a school district competition; when later questioned, the students tended to lie in order to conceal their deceptive transgressions (Fu et al., 2008). Therefore, although children are generally more inclined to tell lies for self-interest as they grow older, they will more often conform to social norms and etiquette in telling prosocial lies in order to maintain social relationships, when costs to self-interest are relatively low (Talwar and Lee, 2008; Lee, 2013). Accordingly, it would appear lying, in addition to being relatively infrequent, is perhaps ironically just as often used for prosocial and social bonding functions, as it is for antisocial deception (Talwar and Lee, 2008).

In addition, further reasons have been presented which argue in favor of a derived function for linguistic deception (Talwar and Lee, 2008). First, it may be unreasonable to assume, at least for antisocial deception, whether lying is especially well-suited to serve the primary adaptive function in children, given their general lack of physical strength and social power (Talwar and Lee, 2008). Second, deception is typically very context-specific; in some situations, lying may be appropriate, but in others honesty is generally a more ideal strategy (Talwar and Lee, 2008; Lee, 2013). Third, with respect to potential adaptive functions for language, considerable evidence shows deception often occurs through non-verbal expressive control in a variety of deceptive situations, demonstrating language is not always a necessary requirement (Talwar and Lee, 2008; Lee, 2013).

Nevertheless, some have suggested a harsh disciplinarian approach to parenting may be directly related to the development of deception in children (e.g., in order to conceal transgressions), as an adaptive response to avoiding severe punishment (Talwar and Lee, 2008). However, given lying typically occurs in many varieties, including antisocial and prosocial lying (e.g., white and blue lies) with prosocial lying occurring later in development, it seems a social bonding function could as legitimately be posited for many uses of deceptive language, as an antisocial function (Talwar and Lee, 2008). Indeed, several studies have shown an increase in antisocial lying in children and adolescents with significant behavioral problems, suggesting a lack of normative development in these cases (Li et al., 2011; Lee, 2013). In summary, studies from developmental psychology similarly indicate an underlying rarity, tendency toward prosociality, and context-sensitive aspect of most cases of lying and deception.

### Human Social Behavior

Studies of human social behavior have further shown that deception is a relatively infrequent occurrence in everyday adult social interactions (Hancock et al., 2004; Serota et al., 2010). For instance, in a sample taken of over a 1000 United States citizens, the mean number of lies told on an average day for a typical individual was less than two (Serota et al., 2010). In addition, the statistical distribution of the population was highly skewed. Of the total lies spoken, 23% were told by one percent of the sample and 50% were told by a mere five percent of individuals tallied. Experiments conducted under controlled laboratory conditions have uncovered very similar findings (Abeler et al., 2012). In fact, analysis of typical human conversational content reveals social relationship topics dominate over two-thirds of total daily conversation time (Haviland, 1991; Dunbar et al., 1997). As in any biological system that has significant time and energetic costs as well as adaptive benefits (Barrett et al., 2002), this certainly begs the question as to why we spend most of our time in the prosocial use of language, if language reputedly evolved primarily for manipulation and deception.

Nonetheless, given so many apparent benefits of deception, often in the interest of manipulating competitors (Dawkins and Krebs, 1978; Byrne and Whiten, 1990; Hauser, 1992), many examining the evolution of language have queried how language remains predominantly honest in the average daily use of language (Knight, 1998; Nettle, 1999; Fitch, 2010). A plausible explanation, suggested by several authors, concerns the potential threat of inauspicious social reputation (Silk et al., 2000; Donath, 2008), in the best case, or implementation of strict social sanctions against deception, in the worst case (Clutton-Brock and Parker, 1995; Lachmann et al., 2001; Bliege Bird and Smith, 2005). Preliminary support for this proposal suggests this may in fact be in the case. For example, a study of American dating over the Internet found that approximately 90% of males and 75% of females were dishonest about at least one physical characteristic in their online personal ads; males were inclined to claim greater height, females lesser weight (Toma et al., 2008). Discrepancies from fact were generally quite small in magnitude, however, suggesting that people were at least partially aware of the potential social repercussions of being identified as deceitful (Toma et al., 2008).

Finally, computational modeling experiments of human social networks demonstrate that deception can be used just as often for prosocial as for antisocial functions (Iñiguez et al., 2014; Barrio et al., 2016). For instance, one study showed that antisocial lies cause networks to become increasingly disrupted, whereas prosocial ‘white’ lies can be beneficial in smoothing the flow of complex social interactions and facilitating larger, more integrated networks (Iñiguez et al., 2014). Moreover, these results further suggest that the capacity to lie in moderation likely emerged after language had already come into existence, as the impact of lying is probably too destructive of social bonding and community solidarity to allow its exclusive evolution (Iñiguez et al., 2014; Barrio et al., 2016). In summary, studies of human social behavior further suggest that instances of dishonest language use are relatively rare, with a more common tendency toward prosociality and context-dependence as the basis for most acts of lying and deception.

## Discussion

Unraveling a comprehensive understanding for language function and evolution is unlikely to be a simple or straightforward process. More plausibly, fully developed modern language is likely to be the result of many different selective processes acting in sequence or aggregation. For instance, given a sufficient opportunistic window, sexual selection in particular often embellishes traits originally provided by more conventional processes of natural selection. Indeed, the stratification of several different selection processes is not unusual in evolutionary biology. Nevertheless, thorough review of the utility of deceptive communication in non-human animal societies, deceptive language use during childhood development, and adult use of deceptive language in human social life suggests a derived function of language for deception. In summary, this analysis suggests a direct functional role for language is most likely to have been associated with social bonding, with occasional outlets for derived uses of deception in context-specific social environments.

A potential objection to the conclusions found here is that language is unlikely to have evolved for a single primary function, as language is so clearly useful for so many different aspects of human social life. Certainly, it is undeniable that language can be pivotal for many different purposes, but usefulness does not equate to a selective pressure or an evolutionarily stable strategy (ESS). The current article in fact maintains that language likely evolved directly for social bonding (Dunbar, 2003, 2004; Roberts, 2010, 2013; Freeberg et al., 2012; Dávid-Barrett and Dunbar, 2013) and secondarily derived for several other functions (De Backer and Gurven, 2006; Galantucci and Roberts, 2014), among them including deception. Indeed, it is a truism within non-human animal communication research that most forms of vocal learning likely originated and evolved for one or at most a few primary selective functions, but not for every conceivable function (Nowicki and Searcy, 2014). Therefore, consistent with conventional evolutionary theory, the claim that language evolved for all functions is both unfalsifiable and biologically improbable. More importantly, however, of the evidence reviewed here, there is very little data to support this claim.

In addition, apart from the extensive experimental data reviewed here in support of deception as a derived function for language, there are additional theoretical reasons which reinforce this conclusion. Indeed, while deception accurately characterizes specific kinds of animal signaling in particular contexts, there are compelling reasons to question whether it can be distinguished as the primary function for the greater part of biological communication systems, including human language. Namely, behavioral ecologists have argued theoretically that Dawkins and Krebs (1978) and Krebs and Dawkins (1984) signal evolution models (which argue that signallers evolve to manipulate receivers, while receivers evolve to resist manipulation) are in fact gravely problematic; if there is, on average, no beneficial information to the receiver of a signal, then receivers should evolve to discount the signal (then signaling ultimately has no benefit to the signaller and the system of communication disintegrates; Searcy and Nowicki, 2005, p. 8).

Granted, an incomplete theoretical explanation to this puzzle was suggested by Zahavi (1975), albeit confined primarily to mate choice or aggressive signaling contexts. More specifically, while males may be disposed to manipulate or deceive females or other male competitors with respect to their biological fitness, these males are actually ‘handicapped,’ thereby confirming the honesty of the signal (Searcy and Nowicki, 2005, p. 9). The prototypical example often given is the large tail of peacocks, as the peacock pays a cost as an assurance of its honesty: only those peacocks of adequately high health, vitality, vigor and quality can afford the most exuberant, burdensome tails in the face of continuous predation (Zahavi, 1975; Grafen, 1990; Zahavi and Zahavi, 1997). Clearly, then such models seem to apply only in a relatively limited number of contexts, and it is further uncertain as to whether these animal models can be indiscriminately applied to human language. Therefore, functionally deceptive signaling is believed to be uncommon, as well as have a generally low cost for the misled; otherwise, the signal would simply be ignored and over time become ineffectual (Fitch and Hauser, 2002; Searcy and Nowicki, 2005). Finally, even strong advocates of this proposal have conceded that prosocial communication is likely to be favored in social interactions requiring trust where group members frequently interact over a long-term basis (Krebs and Dawkins, 1984), as is characteristic of humans and most non-human primates (deWaal, 2005). In other words, lying is only an effective strategy when individuals have reasonable confidence they are ordinarily being told the truth; that is, where previous relationships of social bonding and trust have been built based on language already in place.

## Conclusion

Despite a steadily increasing amount of evidence that language evolved primarily for facilitating social bonding in large and complex social groups (Dunbar, 1993, 2003, 2004; Nettle and Dunbar, 1997; McComb and Semple, 2005; Mesoudi et al., 2006; Roberts, 2010, 2013; Ireland et al., 2011; Weaver and Bosson, 2011; Cohen, 2012; Freeberg et al., 2012; Laidre et al., 2012; Dávid-Barrett and Dunbar, 2013; Redhead and Dunbar, 2013; Galantucci and Roberts, 2014; Pietraszewski and Schwartz, 2014), there is currently no broad consensus as to how language originated in the human species (Számadó and Szathmáry, 2006; Fitch, 2010). Despite the fact that upon closer examination, the deceptive use of language reveals a more direct role for social bonding, and suggests a derived role for deception, there are nevertheless several adherents to the perspective that the primary function for language is deception (Dawkins and Krebs, 1978; Krebs and Dawkins, 1984; Scott-Phillips, 2006). On the contrary, it is likely that some combination of deceptive and honest communication led to the social bonding and mediation effects of language, although the relationship and interplay between the two strategies remains to be thoroughly investigated. Moreover, language probably serves several other important secondary functions (Millikan, 1984; Origgi and Sperber, 2000), among them cultural transmission, pedagogy, cooperation, and sexual advertisement, as well as deception (Számadó and Szathmáry, 2006). Future studies should focus on further theoretical and experimental work which explores how these more subordinate functions interact within the context of social bonding.

## Author Contributions

The author confirms being the sole contributor of this work and approved it for publication.

## Conflict of Interest Statement

The author declares that the research was conducted in the absence of any commercial or financial relationships that could be construed as a potential conflict of interest.
